# Lessons learned from the COVID-19 response in Sri Lankan hospitals: an interview of frontline healthcare professionals

**DOI:** 10.3389/fpubh.2023.1280055

**Published:** 2023-12-06

**Authors:** Nimali Lakmini Munasinghe, Gerard O'Reilly, Peter Cameron

**Affiliations:** Faculty of Medicine, Nursing and Health Sciences, School of Public Health and Preventive Medicine, Monash University, Melbourne, VIC, Australia

**Keywords:** COVID-19, healthcare professional, hospital, pandemic, Sri Lanka

## Abstract

**Introduction:**

The COVID-19 pandemic revealed the lack of preparedness in health systems, even in developed countries. Studies published on COVID-19 management experiences in developing countries, including Sri Lanka, are significantly low. Therefore, lessons learned from pandemic management would be immensely helpful in improving health systems for future disaster situations. This study aimed to identify enablers and barriers to COVID-19 management in Sri Lankan hospitals through healthcare workers’ perceptions.

**Methods:**

Frontline doctors and nurses from different levels of public hospitals were interviewed online. Both inductive and deductive coding and thematic analysis were performed on the transcribed data.

**Result and discussion:**

This study identified four themes under enablers: preparing for surge, teamwork, helping hands and less hospital-acquired infections. Seven themes were identified as barriers: lack of information sharing, lack of testing facilities, issues with emergency equipment, substandard donations, overwhelmed morgues, funding issues and psychological impact. These preparedness gaps were more prominent in smaller hospitals compared with larger hospitals. Recommendations were provided based on the identified gaps.

**Conclusion:**

The insights from this study will allow health administrators and policymakers to build upon their hospital’s resources and capabilities. These findings may be used to provide sustainable solutions, strengthening the resilience of the local Sri Lankan health system as well as the health systems of other countries.

## 1 Background

The COVID-19 pandemic has resulted in a global health and economic crisis (1), emphasising how important it is to be prepared for disasters. The COVID-19 virus was first detected in the city of Wuhan in Hubei Province, China, in late December 2019, and the World Health Organization (WHO) declared COVID-19 a pandemic on 11 March 2020 (2). According to data, COVID-19 has been reported in 539 million people and resulted in over 6.3 million deaths globally by 21 June 2022 (3).

The COVID-19 pandemic has placed a tremendous burden on healthcare systems. The pandemic has also illustrated the risks of global overdependence on a single nation (such as China or India) for essential medicines and medical equipment (4). Even developed countries had to struggle to control the infection and to reduce deaths. Unlike in other disasters, developing nations became helpless without support from developed nations because COVID-19 was a global pandemic. Healthcare workers (HCWs) were at great risk of becoming infected through occupational exposure (5, 6). Thousands of HCWs had been infected and a significant number had died across the world (5, 7, 8). Therefore, protecting HCWs was also a priority in reducing the burden on hospitals.

The fear of devastating impacts continued to grow in developing countries. The lower-income countries and countries with less-resourced health institutions have found it disproportionately hard to expand existing capacity for increasing demand (9). The rapid spread of the infection has overburdened health systems of these countries in terms of critical care provision, including beds in intensive care units (ICUs), mechanical ventilation, supplementary oxygen and the ability to protect HCWs (9).

Developing countries in Asia had faced many challenges in their socioeconomic and healthcare systems (10). Although almost all countries in the world had faced challenges because of COVID-19, South Asian countries in particular dealt with enormous challenges. These were mainly the result of their large population, less-resourced health facilities, high poverty rates and low socioeconomic conditions (10). The COVID-19 pandemic rapidly overwhelmed the fragile health systems of these countries (10). For example, India had periods of COVID-19 crisis for several months in 2021 where hospitals were overcrowded with patients, running out of oxygen and capacity was exceeded (11). The government collaborated with nationwide local authorities to combat the situation to ensure adequate hospital beds, oxygen and anti-viral drugs (12). Because of the surge in COVID-19 deaths, hospital morgues and crematoriums were also overwhelmed. Bodies were piled up and some were cremated in family backyards or even in the streets, while some corpses were thrown into rivers (13).

Studies have also reported that the inefficient management of logistics chains, lack of human resources and inadequate laboratory facilities compromised the readiness of healthcare systems in neighboring countries such as Nepal, Bangladesh and Pakistan (14–16).

As a developing country also situated in South Asia, Sri Lanka was also at risk of experiencing a critical situation with the onset of the pandemic. Early preventive strategies taken by the government and the Ministry of Health (MoH) were considered successful in preventing widespread community transmission (17). However, the subsequent second and the third waves of the pandemic put an increased strain on the local health system, overwhelming its capacity (18). From the first reported case on 27 January 2020 to the end of September 2020, there were only 3,363 confirmed cases and 13 deaths over a period of less than 9 months (17). However, by 26 February 2022, the corresponding numbers had increased to 643,072 positive cases and 16,142 deaths (3). It was inevitable that the local health system would be stretched to its maximum capacity and face challenges. Lessons learned from past disasters are of paramount importance when improving health system resilience. However, there has been a scarcity of research on COVID-19 experience in Sri Lankan hospitals.

As frontline responders, doctors and nurses who were actively involved in COVID-19 management in local hospitals were the ideal personnel to explore hospital preparedness to the pandemic. Therefore, this study aimed to identify enablers and barriers to the COVID-19 response in Sri Lankan hospitals through frontline HCWs’ experiences. These findings would be helpful in improving the resilience of the Sri Lankan health system to future disasters. The lessons would also be useful in providing sustainable solutions for strengthening the resilience of health systems in other countries.

## 2 Methodology

### 2.1 Study design, sampling, and recruitment

A descriptive qualitative approach was used in this study. Because the public health sector is providing universal free health coverage to all citizens in Sri Lanka, almost all COVID-19 cases were managed in public hospitals except for a few cases managed in the private sector. To ensure a generalized understanding, doctors and nurses employed in public sector hospitals in Sri Lanka were recruited, representing both male and female respondents from different categories of hospitals (national hospitals, teaching hospitals, district general hospitals, base hospitals and military hospitals) in different provinces of the country. A snowball sampling technique was employed, leading to the recruitment of different categories of frontline HCWs, representing health administrators (healthcare professionals in administrative roles), consultant physicians, consultant emergency physicians, in-charge nurses and nursing officers.

It was considered that theme saturation would be achieved at approximately 15 interviews which also aligned with the investigators’ intention to minimized additional burden to this already busy period for frontline health care workers. Therefore, recruitment email requests were sent to 20 doctors and nurses explaining the purpose and the manner of the study and also requesting their consent for recording the interview. Eighteen agreed to participate in the study by replying to the invitation email.

### 2.2 Inclusion criteria

Participants were eligible if they were frontline HCWs (doctors and nurses) who had at least 1 month of experience managing COVID-19 patients and more than 5 years of career experience in public sector hospitals and were willing to be interviewed.

### 2.3 Exclusion criteria

All private and public sector hospitals that did not have a COVID ward were excluded from the recruitment procedure.

### 2.4 Data collection

The interviews were arranged for a preferred time and date for each participant during their non-work time. All the interviews were conducted online via Zoom from 15 December 2021 to 15 January 2022. Each interview lasted for between 45 and 60 min. The interviews were continued until data saturation was achieved. Altogether, 16 participants were interviewed, at which point data saturation was confirmed. All 11 doctors were interviewed in English and all five nurses were interviewed in the Sinhala language for their convenience.

These interviews were conducted following a semi-structured, interview guide prepared by the researchers. The guide was based on the 4S domains of hospital disaster preparedness: space, stuff, staff and systems (19–21) (Appendix I). It also focused on identifying demographic characteristics of the respondents along with the identification of enablers and barriers to COVID-19 management.

All the interviews were audio-recorded, with the informed consent of the participants. Confidentiality of the data was ensured. All the interviews were conducted by NM (main author), who had previous experience of conducting interviews. The details of the qualitative interview were reported using the Consolidated Criteria for Reporting Qualitative Research (COREQ) guide (22) (Supplementary Appendix II).

### 2.5 Data analysis

Content analysis was conducted according to the Graneheim and Lundman technique (23). Both inductive and deductive coding and thematic analysis of transcribed data were performed using Microsoft Word.

### 2.6 Rigor or trustworthiness

The trustworthiness of this qualitative study was achieved by ensuring credibility, transferability, dependability and confirmability (24, 25). Several enhancing strategies were used in the study. To enhance credibility, a diverse group of participants was recruited, including both males and females, with different expertise, from different levels of hospitals, representing several districts in the country. The interview guide was designed by NM and GO and revised by a qualitative research expert. The guide was pilot-tested for clarity and comprehension by interviewing a separate doctor and a nurse in Sri Lanka who did not participate in the study. The interviews were transcribed by a professional transcriber who is fluent both in English and Sinhala. The transcripts were verified for accuracy. All the recordings were listened to carefully numerous times; they were then double-checked for clarity by two independent reviewers (NM and GO). The English transcripts of the nurses’ interviews, which were conducted in Sinhala, were checked for clarity by NM, who is fluent in English and whose mother language is Sinhala. All the transcripts were read through several times and coded by two independent reviewers (NM and GO). If any discrepancies occurred, consensus of a third reviewer (PC) was taken to make the final decision. All the codes, categories and themes developed by the independent reviewers were refined by the third reviewer (PC).

To enhance confirmability, member checking was performed, and all the transcripts were sent back to the participants for their opinions and verification of the accuracy of the codes and interpretations. While reporting, evidence was provided using verbatim quotations from the participants.

To enhance dependability, NM made reflexive notes throughout the study, while GO and PC served as auditors and carefully examined the process.

To enhance transferability, the study context was accurately described with the details of participants, sampling methods and procedures of data collection, etc.

### 2.7 Ethics statement

Ethical approval was obtained from the Monash University Human Research Ethics Committee on 12 October 2021 (Project ID: 29716). Administrative approval was obtained from the MoH, Sri Lanka on 8 October 2021.

## 3 Findings

The demographic characteristics of the 16 participants are illustrated in Table 1. There were 10 male and six female respondents from different levels of hospitals, namely, the national hospital, two teaching hospitals, four district general hospitals, four base hospitals, the police hospital and two military hospitals, the Army and the Navy. Their career experience ranged from 11 years to 29 years. Almost all had worked on frontline services from the beginning of the pandemic in Sri Lanka; therefore, they had an average of about 18 months’ experience by the time of the interview. There were five nursing professionals, including two nursing officers and three in-charge nursing officers. There were 11 doctors, including three consultant emergency physicians, five medical administrators, two consultant physicians and one public health specialist (Table 1).

**Table 1:** Demographic characteristics of the respondents.

Category	Sub-category	Number (percentage)
Gender	Male Female	10 (62.5%) 6 (37.5%)
Expertise	Medical administrator Emergency physician In-charge nurse Nursing officer Consultant physician Public health specialist	5 (31.3%) 3 (18.8%) 3 (18.8%) 2 (12.5%) 2 (12.5%) 1 (6.3%)
Work experience (years)	10–14 15–20 21–25 26–30 31–35	4 (25%) 5 (31.3%) 5 (31.3%) 1 (6.3%) 1 (6.3%)
COVID-19 management experience (months)	9–12 13–16 17–20	3 (18.8%) 3 (18.8%) 10 (62.5%)
Hospital type	National Teaching District general Infectious disease Base Army Navy Police	2 (12.5%) 2 (12.5%) 4 (25%) 1 (6.3%) 4 (25%) 1 (6.3%) 1 (6.3%) 1 (6.3%)

The analysis yielded 47 codes, 9 categories and 11 themes. Four themes were identified as enablers of the COVID-19 response in Sri Lanka, namely, preparing for surge, teamwork, helping hands and less hospital-acquired infections. These four themes comprised a total of nine categories. Seven themes were identified as barriers, namely, lack of information sharing/communication challenges, lack of testing facilities, issues with emergency equipment, substandard donations, overwhelmed morgues, funding issues and psychological impact on HCWs (Table 2). The ‘psychological impact on HCWs’ theme will be discussed in a separate paper because of the relatively large amount of content pertaining to this barrier (i.e., this theme resulted in multiple codes and categories from the participant interviews). Therefore, this theme is not included in Table 2.

**Table 2:** Identified codes, categories and themes of enablers and barriers.

Codes	Categories	Themes
Enablers		
1. Modified triage 2. Cancel clinic, OPD, routine surgeries 3. Modified rosters	1. Changes in routine procedures	Preparing for surge
4. Transformed spaces 5. Improve oxygen supply 6. Improve ICU/HDU facilities	2. Infrastructure development	
7. On site/online training 8. Training of trainers	3. PPE training for emergency staff	
9. COVID Cell meetings 10. Key stakeholders’ discussion 11. Decision-making as a team	4. Regular meeting	Teamwork
12. No demarcations 13. Supporting each other 14. Take the risk 15. Sacrifices	5. Dedicated staff	
16. Volunteering 17. Construction of ICC and wards 18. Support transportation 19. Disposal of bodies	6. Military support	Helping hands
20. Donate PPE and medical equipment 21. Sending food for patients/staff 22. Providing dry rations	7. Community support	
23. Frequent sanitising 24. Wearing mask/PPE 25. Hand washing	8. Strictly followed precautions	4. Less hospital-acquired infection
26. Government’s vaccination efforts 27. Priority for health staff	9. Successful vaccination	
Barriers		
28. Multiple reporting 29. Lack of timely update 30. Lack of IT facilities 31. Delayed and poor information sharing		5. Lack of information sharing
32. No PCR machine 33. Few standard laboratories 34. Delayed result		6. Lack of testing facilities
35. Shortage of equipment 36. Poor knowledge of functionality 37. Shortage of consumables		7. Issues with emergency equipment
38. Used items 39. Broken items 40. Hidden agendas of donors		8. Substandard donations
41. Lack of mortuary staff 42. Lack of morgue capacity 43. Problems with cremation		9. Morgue capacity exceeded
44. No accountants 45. Inability to manage monetary donations 46. Prolonged time for processing 47. Lack of funds		10. Funding issues

HDU, high dependency unit; ICC, intermediate care centre; ICU, intensive care unit; IT, information technology; OPD, outpatient department; PCR, polymerase chain reaction; PPE, personal protective equipment.

The data analysis identified the enabler themes described in the following sections.

### 3.1 Preparing for surge

To accommodate a large number of COVID patients, all the hospitals needed to expand available space, the number of beds and other critical care capacities. Three categories were identified under this theme: (i) changes in routine procedures, (ii) rapid development of infrastructure and (iii) training on personal protective equipment (PPE) donning and doffing.

#### 3.1.1 Changes in routine procedures

All the hospitals made considerable changes in routine hospital operational procedures, such as canceling clinics, outpatient department (OPD) and routine surgeries. Triage protocols were modified and treatment protocols and management guidelines were also adopted based on the technical guidance issued by the MoH and the WHO. These changes also included the provision of hand-washing and sanitising facilities at the entrance of all the units, limitation of the number of visitors, maintenance of physical distancing and frequent cleaning of the hospitals.

An administrator from a national hospital described the changes that occurred in his hospital:

We discharged non-urgent patients, cancelled elective surgeries, stopped outpatient treatments and routine clinics. However, we established a system to send the drugs to clinic patients by post, ensuring their safety and providing continuous supply of medicines. We limited the point of entry to the facility. Further, we modified our standard triage system as well. We prepared a general roster for COVID management including all the doctors in the hospital.

#### 3.1.2 Rapid development of infrastructure

All the hospitals rapidly converted some of their wards, transformed existing spaces or abandoned wards for COVID treatment areas and isolation facilities. The ICUs and high dependency units (HDUs) were newly built or upgraded, if already existing. The government provided the necessary logistics and financial resources to these hospitals through the MoH. Further, these hospitals were supported by external donations, and manpower was increased by the voluntary participation of military personnel and the public.

An emergency physician from a district general hospital explained:

Initially, it was a big challenge for us to allocate existing ICU beds to manage COVID patients. But we had a separate hospital, which was not open for the public at that time. Its ICU was not functioning. With the ministerial approval, we established a separate ‘fever corner’, an isolation area and a six-bedded ICU. Now, it is fully dedicated for COVID patients.

A nursing officer from a district general hospital described how they successfully expanded space and bed capacity:

This unit, initially, was a rehabilitation centre. We quickly transformed this into a COVID ward. Gradually, we expanded it up to 300 beds. Then, the ICU was started with three beds. Finally, we could start a treatment centre and a 24 bedded HDU as well.

#### 3.1.3 PPE donning and doffing training

The study participants received PPE training from various platforms, ranging from online videos to hands-on training. The majority of the participants received training at the Infectious Disease Hospital (IDH) in the capital city, Colombo. A small group of frontline doctors, nurses and supporting staff of most of the hospitals were sent to the IDH for a short-term training program on PPE.

A nurse in charge of a COVID ICU in a base hospital described:

We, a team of nurses, sisters and minor staff, had a two-day training at IDH. In addition, our infection control unit also conducted some training for nurses on PPE donning and doffing. The trained staff, then, trained the other staff.

However, some hospitals mostly developed their knowledge and skills through virtual platforms. An emergency physician from a teaching hospital explained how they trained the staff virtually:

We learned, PPE donning and doffing, totally from the online resources like YouTube. Also, we conducted virtual training program for our staff.

### 3.2 Teamwork

All the participants highlighted that during the crisis, everybody worked as a team. The important decisions were made through discussions and regular meetings; everybody supported each other more than ever before. Two categories were identified under this theme, namely, dedicated staff and regular meetings.

#### 3.2.1 Dedicated staff

The respondents highlighted how the frontline workforce was dedicated to their duties by sacrificing the most important life events, social and personal events, the New Year and religious festivals. They were not limited only to their designated duties, but extended full support to cover duties of other categories of staff, when needed. They put their own life at risk to protect others and were fully dedicated to their duties.

An administrator from a military hospital described:

Some members of the staff could not even attend … their mother’s funeral because of … COVID duties. I also had to stay in the East for about 3 months for supervising my team and I was away from my family. We all were fully engaged with our duties.

An administrator from a national hospital described how dedicated their staff was:

It’s very sad to say, in some ICUs all the supporting staff had to be quarantined and all their work had to be done by the nurses and nurses had a very bad time. However, they did a great job. Even though we had loads of challenges, we all worked together towards the goal of saving lives. We did not have any conflicts. So, everybody worked as a team.

A physician from an infectious disease hospital explained:

We had to improvise our own way of doing the ward rounds without exposing the full staff. Normally, we used to do ward rounds with doctors, medical student, nurses and minor staff. But now, I go to the patients alone, to prevent exposing others. Sometimes, I had to take samples for PCR.

#### 3.2.2 Regular meetings

Almost all the respondents described that they had regular meetings with key personnel in the hospital to discuss issues, progress and to make decisions. All the hospitals had a weekly meeting called ‘COVID Cell’; in addition, larger hospitals had daily administrative meetings.

An emergency physician from a district general hospital described:

Actually, we had weekly COVID meetings in our hospital, it’s called COVID Cell and we took most of the critical decisions during this meeting, it’s a multi-disciplinary meeting representing all categories of staff. I represented the emergency department.

A hospital administrator from a teaching hospital explained:

Every morning, we had administrative meetings with the directors, deputy directors, chief nursing officers, accountant and all the key personnel. We were updated with current situations and future plans. We also had twice a week meeting with physicians, surgeons and in charge nurses. All these meetings gave us the strength and we felt that we were together.

### 3.3 Helping hands

The COVID response of most of the hospitals, as well as the national response, was highly supported by the military forces and the public. Two categories were identified under this theme: support from the military and community support.

#### 3.3.1 Support from the military

All the respondents highlighted the contribution of the military to the national response, mainly through the COVID Task Force. Specifically, Army personnel contributed greatly to infrastructure establishment and maintenance of intermediate care centres (ICC) and treatment centres. They also supported the vaccination program, providing voluntary medical teams for some hospitals.

An administrator from a military hospital explained:

We had full deployment of all the staff and they were working 24/7. We provided manpower to the civilian hospitals and heavily involved in vaccinations. As most of the ICC were in faraway places, we had to transport patients and the bodies. The COVID Task Force is also headed by the commander of the Army. The coordination of the health sector and all the other sectors were handled by this task force. Also, almost all the quarantine process was mainly handled by the army, with the support of Air Force and the Navy. Most of the COVID treatment facilities were also developed by Military Engineering Troops.

#### 3.3.2 Community support

All the participants highly valued and appreciated the various forms of support given by the community.

An emergency physician from a district general hospital described how the community helped the hospital:

Our health system is entirely free of charge but compared with the health budget allocated by the government, it is impossible to cater all the facilities for this surge of patients. However, we received donations from the community including medical equipment, PPE, oxygen supply systems worth millions, also dry rations, cloths, food and sanitary items. Sometimes, they provided meals for our staff. Without these donations I do not think we could battle this pandemic.

### 3.4 Less hospital-acquired infection

The other enabler identified was the smaller number of hospital-acquired infections among the staff. Two categories were identified under this theme as contributing factors for success, namely, strictly following precautions and successful vaccination.

#### 3.4.1 Strictly following precautions

All the respondents highlighted that all categories of HCWs carefully followed the precautionary measures. Even though there was a lack of PPE at some stages of the pandemic, they made sure to protect themselves, paying for the cost of PPE by themselves.

A nursing officer from a district general hospital described:

We are directly contacted with the infection, but most of us did not contract the disease. We were very keen on our protection and strictly followed the precautionary measures. Frequent hand washing, sanitising, proper wearing of PPE helps to reduce the transmission of infection among the staff. Only a very few staff members become positive in our ICU, but the origin of the infection was found to be outside sources, not from the hospital.

#### 3.4.2 Successful vaccination

These respondents also appreciated the government initiatives for successful vaccination programs, which involved expediting the procurement process and improving vaccine administration, with the support of the military medical teams.

A nursing officer from a base hospital explained:

Actually, the vaccination program of the country was very successful. Health workers were given priority and we have already completed three doses of Pfizer. Now, we feel like we are fully immune. Therefore, the staff aren’t scared to work as before.

The data analysis identified the barriers (not including the theme, psychological impact on HCWs) described in the following sections.

### 3.5 Lack of information sharing/communication challenges

Participants experienced a lack of timely updates on information related to COVID-19. They also found some challenges in accessing that information via online platforms because of several reasons, including the lack of a user-friendly website and the lack of communication infrastructure and IT facilities.

An administrator from a base hospital described:

Poor information sharing was a main problem. There was much delay in receiving management protocols from the Ministry. It was difficult to trace the latest version of the protocol from the Ministry website as the indexes were not updated. Also, we had to prepare multiple reports to inform our daily statistics to several institutions. This reporting was an extra burden with the limited staff and lack of IT facilities.

A nursing officer expressed the lack of IT facilities in a district general hospital:

We do not have a computer in our ICU, no WhatsApp facilities or other IT facilities for us to communicate. When the director’s office is closed, we have to wait until it opens, even to get a printout.

### 3.6 Lack of testing facilities

Initially, most of the hospitals had challenges with testing facilities. They had to send samples to Colombo or to laboratories in other locations. Those labs were overwhelmed with samples from all over the country. Therefore, it took an average of 3 to 5 days to obtain the result. However, later, most of the hospitals received polymerase chain reaction (PCR) machines and other test kit facilities to overcome this barrier.

A nursing officer from a district general hospital explained:

Actually, we had to face a lot of trouble even to do our PCRs. We did not have a PCR facility and had to send the sample to Karapitiya. It takes about 4 days to get the result.

An administrator from a base hospital described:

Initially, we had to take the samples and send to whatever the available laboratory. Some laboratories sent the reports very late. After few months, we got a PCR machine.

### 3.7 Issues with emergency equipment

At the initial stage, almost all the hospitals experienced a shortage of emergency equipment. Even though the MoH provided them with some equipment, it was not sufficient to manage the huge influx of patients. The community also donated much equipment; however, this was not enough. Some hospitals received adequate equipment, but they did not receive proper guidance or training on the functionality and maintenance of the equipment (“poor knowledge of functionality” to be interpreted as suboptimal training / orientation / in-service in the use of emergency equipment). Further, the participants highlighted that some equipment could not be used because of the unavailability of consumables. Conversely, respondents from the police and military hospitals reported having an adequate amount of emergency equipment, including PPE and other essential supplies.

A nursing officer from a base hospital described:

We received a lot of monitors, CPAP (continuous positive airway pressure) and Bi-PAP (bilevel positive airway pressure) machines as donations. Only the ETU (Emergency Treatment Unit) staff had training on this equipment. Nurses from different units were allocated for each shift at the COVID wards and they were not trained. Therefore, the majority had poor knowledge on the functionality and the protection of the equipment.

A nursing officer from a district general hospital described why some equipment became unusable:

The business community and well-wishers have given us many equipment worth millions, ventilators, syringe pumps, high flow machines, defibrillators etc. However, some equipment could not be used because of lack of parts to replace, for example, we have defibrillators with sticky pads, these pads are not available in the country because of increased demand.

### 3.8 Substandard donations

Some interviewees complained about donations. Some donations were substandard, while some were purely for publicity. This created an unnecessary burden for the administrators.

An administrator from a base hospital described:

Some community donations were either used or not having service agents in the country. Some were broken and just piling up as garbage in our hospital. As we must keep the inventories of all the donations, getting rid of them is an extra burden. The Ministry should regulate such poor-quality donations.

Another administrator from a different base hospital expressed:

Some donations were tricky, because some people donated a few items and they just wanted to get huge publicity. They needed to take photos and videos and put them on social media or other media for publicity. This type of donation makes unnecessary trouble for us.

### 3.9 Morgue capacity exceeded

The majority of the participants described that they could manage the morgue at their hospital through the rapid cremation of bodies within 24–48 h. However, a few hospitals faced a situation where morgue capacity was exceeded.

An administrator from a base hospital described how the morgue of his hospital was overwhelmed.

We got several bodies from the community, but we have only four coolers. We were overwhelmed by home deaths. A negative PCR was needed if a post-mortem was required on a death. Initially, we did not have a PCR machine. So, it took several days to get the report. So, we had to keep the body outside for about a day, once a cooler was available, we put them into the freezer. Sometimes, bodies were decomposed partially and we were blamed. Anyway, we had to care for the living rather than the bodies.

The same hospital faced a serious problem because of overstretched morgue capacity.

We had an issue with the swapping of two bodies; the relatives were given the wrong bodies. This happened because of a checking mistake of one of the minor staff members and the supervising person. This was partially because of the problem with our staff and also because of the overwhelming of our morgue capacity. This became a big issue and was highlighted in all the media.

Another emergency physician from a teaching hospital described:

Actually, our mortuary capacity was exceeded and we had to keep the bodies outside the mortuary for about 1 day. However, the army personnel assisted us to quickly remove the bodies, arranging cremations.

### 3.10 Funding issues

Financial resources were a great challenge in all the hospitals. Unlike the hospitals governed by the MoH (e.g., the national, teaching and district hospitals), the provincial hospitals, which are governed by provincial ministries, faced many difficulties with financial resources. Respondents described the complicated and time-consuming procedure of the approval of costs at the provincial level. The main barrier to maintain a contingency fund was the unavailability of an accountant in the provincial hospitals.

An administrator from a base hospital described why he could not maintain emergency funds:

We cannot maintain any funds at the base hospital levels as we do not have an accountant. We have to request funds from the RDHS (Regional Director of Health Services) and it takes so much time to process. I did not entertain monetary donations because there is no proper system to manage funds within our type of hospitals.

Another administrator from a different base hospital highlighted the same issue:

Lack of funds is the main challenge in managing a disaster in provincial setup. Even though the line ministry issues funds quickly in emergency situations, the provincial setup takes a long time. I have only 5,000 rupees for my petty cash and 20,000 rupees for emergency drugs. That’s the only funds I have. We do not have an accountant.

## 4 Discussion

To the best of our knowledge, this is the first study in Sri Lanka to examine the enablers and barriers to COVID-19 management through the perceptions of both doctors and nurses in the frontline of the healthcare system. This study identified several enablers and enormous challenges faced by frontline HCWs in battling the ‘perfect storm’ of the COVID-19 pandemic in Sri Lanka. The study revealed four main themes of enablers of the COVID-19 response: preparing for surge, teamwork, helping hands and less hospital-acquired infections. Seven themes were identified as main barriers: lack of information sharing/communication, lack of testing facilities, issues with emergency equipment, substandard donations, exceeded morgue capacity, funding issues and psychological impact on HCWs. The last theme was not included in this discussion, as explained earlier.

The Sri Lankan government’s key interventions included the establishment of the National Task Force to coordinate the COVID response; imposing island-wide lockdowns at an early stage of the pandemic; closure of all ports of entry, schools and universities; mandatory face mask-wearing; social distancing measures; intense contact tracing; strict 14-day quarantining and disinfecting public places. These interventions helped keep the first wave in Sri Lanka under effective control (26). The quick establishment of designated quarantine centres with the support of military personnel was also immensely helpful for the public as all the facilities were provided free of charge.

This study reported that the government’s well-organized and coordinated national response was highly successful in controlling disease transmission as well as improving hospital surge capacity nationwide. This response was primarily handled by the MoH, in collaboration with the National COVID Task Force (17). The response was also supported by the WHO, many other private and public organizations and the general public. In preparing for surge, almost all the hospitals successfully converted their existing wards or other available spaces into COVID wards within a short period of time. A reserved pool of military personnel was quickly mobilized and these rapid transformations were supported by voluntary contributions. The government expedited the process by providing necessary logistics and financial support through the MoH. Further, some hospitals established or upgraded their existing ICUs and HDUs. Most of the hospitals developed COVID-19 treatment protocols and management guidelines based on the technical guidelines issued by the MoH and the WHO.

However, frontline HCWs also struggled with multiple challenges because of inadequate preparedness at hospital level. Base hospitals were the smallest hospitals included in this study and they were managed by the provincial ministries. This study highlighted that base hospitals had more difficulties than bigger hospitals because of lack of resources. It was also revealed that these hospitals faced multiple challenges because of lack of efficient financial management systems.

However, the military and the police hospitals were better prepared in terms of all resources compared with other hospitals. This success may be because of the separate governance of these hospitals by the Ministry of Defence, and also because of the better funding system and their more positive mindset thanks to training.

As found in this study, the lack of IT facilities was a huge barrier in communication and information sharing in most of the hospitals in Sri Lanka. In contrast, effective use of technology in the digitalisation of healthcare, such as in contact tracing, surveillance, sharing real-time data, laboratory networking and coordinating with other stakeholders, were key in the successful pandemic management of other countries (27). For example, the South Korean government disclosed real-time COVID-19 information through mass media, dedicated websites, phone messages and mobile apps (28, 29).

Communication is of paramount importance in a disaster response to enable effective coordination and collaboration within the hospital as well as with external stakeholders. Many countries shifted to virtual communication during the pandemic to ensure effective communication, fast learning and knowledge updating (30). Even though people were physically distancing, these platforms kept them more connected socially than ever before. Some countries also introduced telemedicine practices, ensuring the safety of both patient and the practitioner while keeping face-to-face contact. Therefore, the accelerated expansion of telehealth became one of the most important changes in the delivery of healthcare during the pandemic (31).

Further, to draw meaningful insights, it is vital to have timely access to real-time data, especially in a pandemic situation. However, in Sri Lanka, health information systems were found to be inadequate and underfunded. The lack of IT facilities and the lack of an appropriate central health database system hampered health information sharing among hospitals (28). Our study highlighted that the smaller hospitals were most affected by the poor communication of real-time updates on COVID-19.

Sri Lankan hospitals still have a manual documentation system. As highlighted by our study, the preparation of multiple documents and daily reporting to several places were an extra burden for the limited staff, especially with poor IT facilities. In contrast, with the surge of COVID-19 patients, the emergency departments of developed countries adopted multiple electronic health record process improvements to reduce the burden of documentation (32). Other countries also provided technologies to facilitate communication between patients and their families by video conferencing (32). They found this to be beneficial for both patients and their families.

This study found that the lack of dedicated laboratories and testing facilities severely compromised the efforts of battling the pandemic in Sri Lanka. Improving testing capacities was one of the first priorities to control the spread of the disease in every country. For example, Australia, with a population of 25.4 million, conducted over 63,000 daily PCR tests in June 2020 (33). However, during the same period, for an almost similar population of 21.5 million (34), the total PCR testing capacity of Sri Lanka was 2,526 per day (35).

The WHO recommends that highly infectious samples should be tested at biosafety level (BSL) 3/4 type laboratories (36). These are highly sophisticated facilities that require specialized expertise. At the beginning of the pandemic, there were no such facilities functioning in Sri Lanka. Therefore, the laboratory staff had to conduct COVID testing in high-risk, routine laboratory environments. The Medical Research Institute (MRI), Colombo, established a BSL3 laboratory decades ago; however, it had not been in operation since 2002 (37). Several other BSL2 laboratories are located in medical colleges and universities. The lack of expertise in this field was a major barrier. However, at the beginning of 2020, the MoH had taken initiatives to mobilize resources to implement a BSL3 laboratory at MRI (37). At the same time, the Interim Biosafety Guidelines for Laboratories were issued by the MoH to inform the laboratory staff on safety precautions while handling samples (38). However, Sri Lanka’s ability to fight the pandemic was compromised by limited testing facilities.

This study also identified that local hospitals were short of emergency equipment during the peak of the pandemic; this was similar to many countries around the world. The demand for emergency medical equipment quickly exceeded supply, leading to critical shortages, especially, ventilators and PPE. Some countries adopted creative and timely strategies to overcome this challenge. They relaxed the regulations imposed on manufacturing and promoted local production. As an example, the US Food and Drug Administration provided maximum regulatory flexibility for manufacturing to increase the availability of ventilators, other respiratory devices and accessories (39). This flexibility encouraged manufacturers and increased local production. To increase the number of existing ventilators, these guidelines also recommended that hospitals use ventilators beyond their shelf life and also to use ventilators intended for other purposes (39, 40). For instance, they allowed ventilators normally used at home or during transport to be used in hospitals for the long term. They also advised the use of non-invasive breathing equipment for stable patients. This type of flexibility could be adopted by the Sri Lankan government to improve the availability of emergency equipment in its hospitals. In addition, this study found that substandard donations imposed an extra burden on hospitals.

This study also emphasized the lack of morgue capacity in hospitals. During the peak of the pandemic, hospital morgues across the globe became overwhelmed. However, hospitals in developed countries expanded their morgue capacity using large portable refrigerator units as makeshift morgues (41). Because of the high infectivity, the WHO recommended following certain guidelines when handling COVID-19 bodies (42). The WHO also stressed that morgue staff must be trained to use appropriate PPE, and effort should be made to ensure the timely and reliable identification, documentation and traceability of the dead (42). However, it is questionable whether resource-poor countries could adopt such guidelines. Generally, in such countries, untrained or minimally trained staff handled dead bodies. As revealed in our study, Sri Lanka also experienced some mishaps in the handling of dead bodies because of staff shortages, inadequate morgue capacity and lack of documentation and supervision.

Adequate financing is of paramount importance for maintaining a strong and resilient health system and also for continuing essential health services in any disaster (43). As a middle-income country, Sri Lanka has many socioeconomic problems. In addition, the country’s main income sources were also severely affected by the global pandemic (44). Therefore, the country had to mount its response to the pandemic with limited financial resources. Many capacities that are crucial to preparedness can only be built over time and require sustained commitment and funding (43).

According to our findings, most of the base hospitals experienced financial barriers, and these were largely caused by the inefficient system of financial management at the provincial level. Although decentralization has given the provinces the power to formulate their own statutes in Sri Lanka, there is a high degree of financial dependence on the central government. Certain processes were affected by the additional administrative layers at the provincial level, resulting in unnecessary delays. Therefore, generally, most provincial councils were not as efficient and effective in their service delivery as the central ministry.

Many studies have acknowledged the psychological impact of COVID-19 on HCWs (45–49). However, limited studies have focused on how other aspects of hospital preparedness, such as donations, communication, laboratory facilities and morgue capacity, have affected the COVID-19 response, as examined in our study. Similarly to our findings, a few studies have reported that lack of training, limited PPE, lack of testing facilities and funding were barriers to managing COVID-19 in most of the health systems around the world, including developed countries (50–52).

This study identified that very few HCWs had hospital-acquired infections because of adherence to strict precautionary measures at the hospital level. This is confirmed by a study conducted in a base hospital in Sri Lanka, where only 28% of infected HCWs of that hospital acquired COVID-19 from the hospital setting (53).

A study conducted in the United Kingdom (UK) on the COVID-19 management experience of HCWs reported that the redeployment of staff to ICU duties heightened the feeling of being unprepared as PPE simulation was the only training they received (50). Our participants experienced the same situation because of moving non-trained staff to the emergency department to address severe staff shortages. However, UK staff had opportunities to access online training to improve their capacity. In contrast, the majority of our study participants did not even have free access to the internet, resulting in further barriers to improve their knowledge and skills.

Many countries highlighted the importance of community support received during the COVID-19 response (54–56). Our study also identified community support as an enabler; however, sometimes, the donations became additional burden for Sri Lankan hospitals because of the poor-quality or substandard donations they received. This was a significant finding of our study, which was not reported in other COVID-19 studies. Similar incidents were also reported in Sri Lanka during the tsunami of 2004. The uncoordinated and substandard donations resulted in negative impacts on the relief and recovery process (57).

Studies have also reported that information overload was a common problem experienced by HCWs in other countries (51, 58). In contrast, lack of information sharing was a significant finding in our study context. This may be because of the lack of communication facilities and IT facilities in most of the rural hospitals.

## 5 Recommendations

To ensure that the Sri Lankan health system is more disaster-resistant, the Ministry of Health, health planners, policymakers and hospital administrators are encouraged to take appropriate action with regard to the following;

- Improve communication infrastructure in the hospitals and implement new technologies to enhance information-sharing through appropriate platforms.
- Strengthen the IT systems of local hospitals. Specifically, the healthcare workers in base hospitals and district general hospitals should be provided with internet access.
- Provide regular training and simulation exercises to ensure the capacity building of staff.
- Improve laboratory facilities and train laboratory staff.
- Promote local production of PPE and emergency equipment and improve the quality and availability of these equipment in local hospitals.
- Introduce an appropriate regulatory mechanism to coordinate and monitor donations, and to prevent substandard donations to hospital
- Find ways to expand mortuary capacity and conduct the appropriate training of morgue staff.
- Allocate adequate funding for capacity building, surveillance, information management, risk communication and essential logistics requirements.
- Provide the administration with the required expertise and authority for handling a contingency fund in provincial hospitals.

## 6 Limitations

The participants of this study were restricted to the frontline doctors and nurses in hospitals. Thus, the experience of other categories of frontline HCWs in hospitals, such as paramedics, laboratory staff, mortuary staff, kitchen staff, waste management staff, radiology department staff, etc., were not included in this study. Moreover, community HCWs were not included. The interviews were conducted at one point in time and respondents’ attitudes can change over time.

## 7 Conclusion

The Sri Lankan government’s multi-disciplinary team approach and well-coordinated response to COVID-19 was highly successful in controlling the spread of the disease during the initial stage of the pandemic.

This study identified some positive impacts of the pandemic through a concerted national response, community engagement, support and donations to frontline workers. The HCWs also demonstrated much dedication and unity. An extremely low prevalence of hospital-acquired infections among the health staff was observed, possibly resulting from close adherence to precautionary measures and a successful immunization program.

However, significant gaps were also identified because of the lack of emergency training, inadequate testing facilities, poor information sharing and morgues exceeding capacity. In addition, poor-quality donations imposed an unnecessary burden on some hospitals. Moreover, inadequate IT facilities and communication infrastructure hindered information sharing, communication and access to online resources for the staff in smaller hospitals. Further, because of a lack of funds and a flexible funding management system, the provincial hospitals faced multiple challenges.

Such barriers should be addressed to better prepare hospitals for future disasters. These lessons may serve as a starting point for crafting new plans and recalibrating existing plans. Further research is needed to gain a deeper understanding of the enablers and barriers at different levels of hospitals across the island.

## Data availability statement

The raw data supporting the conclusions of this article will be made available by the authors, without undue reservation.

## Ethics statement

The studies involving humans were approved by the Monash human research ethics committee. The studies were conducted in accordance with the local legislation and institutional requirements. The participants provided their written informed consent to participate in this study. Written informed consent was obtained from the individual(s) for the publication of any potentially identifiable images or data included in this article.

## Author contributions

NM: Conceptualization, Methodology, Data curation, Formal analysis, Writing – original draft. GO: Conceptualization, Methodology, Formal analysis, Supervision, Writing – review & editing. PC: Supervision, Writing – review & editing.
