# Supplementary material for: Lessons learned from the COVID-19 response in Sri Lankan hospitals: an interview of frontline healthcare professionals
Source: Front Public Health. 2023 Dec 6;11:1280055. doi: 10.3389/fpubh.2023.1280055 (PMC10731292; doi:10.3389/fpubh.2023.1280055)
Supplement: Supplementary file 1 [file Data_Sheet_1.docx]

**Appendix I**

**Interview topic guide**

1. Tell me about yourself (probing questions to identify demographic characteristics of the respondents).
2. Gender? Marital status? What is your job title? What is the hospital and department in which you work? What is your role and responsibility? How many years of work experience do you have in your carrier? How long (months of experience) have you managed COVID-19 patients?
3. Tell me about your experience with COVID-19 management at the hospital?
4. What sort of training have you have received specifically on COVID-19? (Probing questions: Have you received any training or updating of knowledge on COVID-19 symptoms, case management, personal protection? Have you been trained on donning and doffing of personal protective equipment (PPE)?
5. What are the greatest challenges you encountered in the pandemic management?
6. What changes in process that have been implemented for the pandemic management? (Probe: How each domain (infrastructure, logistics, human resource and systems) of preparedness changed in your hospital?).
7. What was the impact of the pandemic on people? (Probing questions: What was the impact on you, your routine activities, your duties? How was your family affected? How were your colleagues affected?).
8. What do you think about your hospital’s preparedness for this pandemic?
9. How does your hospital prepare for surges of patients? (Probing questions on expansion of space/infrastructure; ICU beds; ventilators; isolation facilities; morgue facilities; adequacy of logistic supplies, such as drugs, equipment and PPE; human resources; plans, guidelines and protocol procedures).
10. How do you feel about the support given to you by the hospital administration? (Probing questions on supply of PPE, vaccination, duty roster arrangement, transport arrangement, accommodation facilities, psychosocial support, rewards, incentives, overtime payments, etc.)
11. What sort of support did your hospital receive from the community? (Probing questions on volunteers, donations, networking/collaboration with other hospitals and external agencies).
12. What do you think was particularly successful in COVID-19 management?
13. What do you think were barriers to preparedness?
14. What are your suggestions to improve these barriers to handle future disaster situations successfully? (Probing questions: What sort of support would you expect for healthcare workers? How could the hospital’s capacity be improved for rapid response? What changes should be made in plans, policies, guidance and emergency protocols, community engagement?).
15. Do you have any other important facts to be shared with me related to COVID-19 management in your hospital?

**Appendix II**

**COREQ (consolidated criteria for reporting qualitative research) Checklist**

A checklist of items that should be included in reports of qualitative research. You must report the page number in your manuscript where you consider each of the items listed in this checklist. If you have not included this information, either revise your manuscript accordingly before submitting or note N/A.

| **Topic** | **Item no.** | **Guide questions/description** | **Reported on page no.** |
| --- | --- | --- | --- |
| **Domain 1: Research team and reflexivity** |  |  |  |
| *Personal characteristics* |  |  |  |
| Interviewer/facilitator | 1 | Which author/s conducted the interview or focus group? | Page 5 |
| Credentials | 2 | What were the researcher’s credentials? E.g. PhD, MD | Title page |
| Occupation | 3 | What was their occupation at the time of the study? | Title page |
| Gender | 4 | Was the researcher male or female? | N/a |
| Experience and training | 5 | What experience or training did the researcher have? | Page 4 |
| *Relationship with participants* |  |  |  |
| Relationship established | 6 | Was a relationship established prior to study commencement? | Page 3 and 4 |
| Participant knowledge of the interviewer | 7 | What did the participants know about the researcher? (e.g. personal goals, reasons for doing the research. | Page 3 and 4 |
| Interviewer characteristics | 8 | What characteristics were reported about the inter viewer/facilitator? (e.g. bias, assumptions, reasons and interests in the research topic). | Page 4 |
| **Domain 2: Study design** |  |  |  |
| *Theoretical framework* |  |  |  |
| Methodological orientation and Theory | 9 | What methodological orientation was stated to underpin the study? (e.g.  grounded theory, discourse analysis, ethnography, phenomenology, content analysis). | Page 3 |
| *Participant selection* |  |  |  |
| Sampling | 10 | How were participants selected? (e.g. purposive, convenience, consecutive, snowball). | Page 3 |
| Method of approach | 11 | How were participants approached? (e.g. face-to-face, telephone, mail, email). | Page 3 |
| Sample size | 12 | How many participants were in the study? | Page 4 |
| Non-participation | 13 | How many people refused to participate or dropped out? What were the reasons? | Page 4 |
| *Setting* |  |  |  |
| Setting of data collection | 14 | Where was the data collected? (e.g. home, clinic, workplace). | Page 4 |
| Presence of nonparticipants | 15 | Was anyone else present besides the participants and researchers? | N/A |
| Description of sample | 16 | What are the important characteristics of the sample? (e.g. demographic data, date). | Page 6 and 29 |
| *Data collection* |  |  |  |
| Interview guide | 17 | Were questions, prompts, guides provided by the authors? Was it pilot tested? | Page 5 |
| Repeat interviews | 18 | Were repeat inter views carried out? If yes, how many? | N/A |
| Audio/visual recording | 19 | Did the research use audio or visual recording to collect the data? | Page 4 |
| Field notes | 20 | Were field notes made during and/or after the interview or focus group? | Page 5 |
| Duration | 21 | What was the duration of the inter views or focus group? | Page 4 |
| Data saturation | 22 | Was data saturation discussed? | Page 4 |
| Transcripts returned | 23 | Were transcripts returned to participants for comment and/or correction? | Page 5 |
| **Topic** | **Item No.** | **Guide Questions/Description** | **Reported on Page No.** |
| **Domain 3: analysis and findings** |  |  |  |
| *Data analysis* |  |  |  |
| Number of data coders | 24 | How many data coders coded the data? | Page 6 |
| Description of the coding tree | 25 | Did authors provide a description of the coding tree? | Page 30 and 31 |
| Derivation of themes | 26 | Were themes identified in advance or derived from the data? | Page 5 and 6 |
| Software | 27 | What software, if applicable, was used to manage the data? | Page 5 |
| Participant checking | 28 | Did participants provide feedback on the findings? | Page 5 |
| *Reporting* |  |  |  |
| Quotations presented | 29 | Were participant quotations presented to illustrate the themes/findings?  Was each quotation identified? (e.g. participant number). | Page 7-15 |
| Data and findings consistent | 30 | Was there consistency between the data presented and the findings? | N/A |
| Clarity of major themes | 31 | Were major themes clearly presented in the findings? | N/A |
| Clarity of minor themes | 32 | Is there a description of diverse cases or discussion of minor themes? | N/A |

Developed from: Tong A, Sainsbury P, Craig J. Consolidated criteria for reporting qualitative research (COREQ): a 32-item checklist for interviews and focus groups. *Int J Qual Health Care*. 2007;19(6):349­–57.
